# Comparative Efficacy of Oral Tranexamic Acid, Intradermal Tranexamic Acid, and Fractional Er: YAG Laser Combined With Hydroquinone in Refractory Melasma: A Randomized Clinical Trial

**DOI:** 10.1111/jocd.71063

**Published:** 2026-07-12

**Authors:** Eisa Mohamed Hegazy, Moustafa Adam El Taieb, Heba Allah Mohamed Mustafa Mohamed, Mohamed Amer Ahmed Abdellatif, Ebtehal Alaa Kotop, Mahmoud Ahmed Ali

**Affiliations:** ^1^ Dermatology, Venereology, and Andrology Department, Faculty of Medicine Qena University Qena Egypt; ^2^ Dermatology, Venereology, and Andrology Department, Faculty of Medicine Aswan University Aswan Egypt

**Keywords:** Er:YAG laser, hydroquinone, MASI, melasma, tranexamic acid

## Abstract

**Background:**

Refractory melasma is difficult to treat because of its chronic relapsing behavior and complex pathophysiology. Combination treatment may improve outcomes over topical depigmenting treatment alone.

**Objective:**

To compare the efficacy and safety of oral tranexamic acid (TXA), fractional Er:YAG laser, and intradermal TXA, each combined with topical 4% hydroquinone in patients with refractory melasma.

**Methods:**

This randomized clinical trial included 45 patients with refractory melasma who were assigned equally to three treatment groups: oral TXA plus hydroquinone, fractional Er:YAG laser plus hydroquinone, or intradermal TXA plus hydroquinone. Clinical response was evaluated using the Melasma Area and Severity Index (MASI). All groups received background hydroquinone and photoprotection; therefore, the study compares adjunctive treatment strategies rather than isolating the independent effect of hydroquinone.

**Results:**

All groups exhibited a significant improvement in the total MASI score after treatment. Oral TXA showed the greatest reduction from 23.61 +/− 1.9 to 9.30 +/− 1.6, followed by fractional Er:YAG laser from 18.91 +/− 2.1 to 11.56 +/− 1.9 and intradermal TXA from 20.41 +/− 1.7 to 13.48 +/− 2.3. Adverse events were mild and transient, and no thromboembolic events or treatment discontinuations occurred. The largest percentage improvement was achieved with oral TXA.

**Conclusion:**

Oral TXA combined with hydroquinone produced the greatest short‐term MASI improvement in refractory melasma, while fractional Er:YAG laser and intradermal TXA remained useful alternatives. Because follow‐up was limited to 3 months, recurrence rates, durability of response, and long‐term safety require confirmation in longer studies.

**Trial Registration:**

ClinicalTrials.gov registration number: NCT06522984

## 1 Introduction

Melasma is a common acquired hyperpigmentary condition characterized by chronic, relapsing, symmetrical brown to gray‐brown macules and patches primarily involving sun‐exposed areas of the face. It is frequent in Fitzpatrick skin types III‐V and commonly affects centrofacial, malar, and mandibular regions. Although medically benign, melasma can cause considerable cosmetic concern, psychological distress, reduced self‐confidence, and impaired quality of life, especially among women [1, 2, 3].

The pathophysiology of melasma is multifactorial and includes genetic predisposition, ultraviolet and visible light exposure, hormonal influences, pregnancy, oral contraceptive use, oxidative stress, vascular changes, inflammation, and extracellular matrix alterations. These overlapping mechanisms may explain its chronic course, inconsistent response to treatment, and recurrence after discontinuation of therapy [4, 5, 6].

Management of melasma remains challenging. Standard options include strict photoprotection, topical depigmenting agents, chemical peeling, systemic therapies, microneedling, and laser‐ or light‐based procedures. Hydroquinone remains one of the most widely used topical treatments because it inhibits tyrosinase activity and decreases melanin formation. However, hydroquinone alone may be insufficient in refractory disease, and prolonged use may be limited by irritation, post‐inflammatory hyperpigmentation, and recurrence [7, 8].

Tranexamic acid has emerged as a useful treatment option for melasma. It inhibits the plasminogen‐plasmin pathway, thereby reducing ultraviolet‐induced melanocyte activation, inflammatory mediator release, angiogenesis, and melanogenesis. TXA can be administered orally, topically, intradermally, or through assisted delivery techniques. Oral TXA has shown encouraging outcomes, but systemic adverse‐effect concerns, particularly thromboembolic risk in susceptible patients, have increased interest in intradermal delivery [9, 10].

Fractional Er:YAG laser is another therapeutic option for refractory melasma. The 2940‐nm Er:YAG laser is highly absorbed by water‐containing tissues and allows controlled fractional ablation, epidermal regeneration, removal of melanin‐containing keratinocytes, and dermal remodeling when combined with appropriate topical therapy and photoprotection [11].

Despite multiple available treatment options, the optimal adjunctive therapy for refractory melasma remains uncertain. Therefore, this randomized clinical trial compared oral TXA, intradermal TXA, and fractional Er: YAG laser, each combined with topical 4% hydroquinone, in patients with refractory melasma.

## 2 Patients and Methods

### 2.1 Study Design and Setting

This randomized clinical trial was performed from June 2024 to June 2025. Forty‐five patients with refractory melasma were enrolled and randomly assigned into three equal treatment groups using a computer‐generated randomization sequence. Sealed opaque envelopes were used for allocation concealment. Participants were randomized 1:1:1, with 15 patients in each group. Outcome assessors and data analysts were blinded to treatment allocation to minimize assessment bias.

### 2.2 Aim of the Study

The purpose of this study was to compare the clinical efficacy and safety of oral TXA, intradermal TXA, and fractional Er:YAG laser, each combined with topical 4% hydroquinone, in the treatment of refractory melasma.

### 2.3 Eligibility Criteria

Eligible patients were older than 18 years, of either sex, and had a clinical diagnosis of refractory melasma. Refractory melasma was defined as melasma persisting for at least 6 months with inadequate response after at least 3 months of standard topical therapy, including hydroquinone‐based regimens and consistent photoprotection.

Because all participants had previously failed hydroquinone‐based treatment, hydroquinone was used as a standardized background therapy in all groups rather than as a separate monotherapy control. The revised interpretation therefore focuses on the comparative benefit of the adjunctive interventions added to hydroquinone, not on the independent effect of hydroquinone itself. The absence of a hydroquinone‐only control group is acknowledged as an important limitation.

Exclusion criteria included pregnancy, lactation, use of oral contraceptive pills or phototoxic drugs within 1 month before enrollment, history of thrombosis, abnormal bleeding profile, known hypersensitivity to TXA or hydroquinone, and relevant endocrine disorders.

### 2.4 Interventions

Patients were randomized to one of three treatment arms. Group I included 15 patients treated with oral TXA 500 mg once daily for 3 months plus topical 4% hydroquinone cream once nightly on the hyperpigmented lesions. Group II included 15 patients treated with fractional Er:YAG laser sessions at 3‐week intervals plus topical 4% hydroquinone. Group III included 15 patients treated with intradermal TXA injections into melasma lesions every 3 weeks plus topical 4% hydroquinone cream nightly.

All patients in the three groups were instructed to use strict photoprotection with broad‐spectrum sunscreen (SPF > = 50) during the study period. Bland emollients were permitted in all groups when irritation or dryness occurred. In the laser group, emollients were specifically used as part of the immediate post‐procedure care, and hydroquinone was temporarily withheld until complete re‐epithelialization.

### 2.5 Clinical Evaluation

Baseline evaluation included demographic data, duration of melasma, triggering events, treatment history, Fitzpatrick skin type, and melasma pattern. Standardized clinical photographs were obtained at baseline and after treatment using the same camera, fixed lighting conditions, an identical background, and consistent patient positioning to ensure a valid visual comparison.

### 2.6 Ethical Considerations

The investigation was performed in accordance with the ethical principles of clinical research. All participants provided written informed consent before enrollment, including consent for treatment, clinical assessment, and use of de‐identified clinical images when applicable. The study was approved by the Institutional Review Board of the Faculty of Medicine, Aswan University, Aswan, Egypt (IRB code: 932/6/24) and was registered at ClinicalTrials.gov (NCT06522984).

### 2.7 Intervention Protocols

Fractional Er:YAG laser treatment was performed using a Fotona 2940‐nm Er:YAG laser system with a fractional ablative handpiece. Treatment was tailored to lesion severity and patient tolerability with energy levels of 400 to 600 mJ per microbeam, a treatment density of 5% to 10%, a 7‐mm spot size, and one to two passes per session. Three sessions were conducted at 3‐week intervals. Topical 5% lidocaine cream was applied under occlusion 30 to 45 min before each session. The planned clinical endpoint was homogeneous erythema without localized hemorrhage. Bland emollients and strict photoprotection with broad‐spectrum sunscreen (SPF > = 50) were used for post‐procedure care. Topical 4% hydroquinone was resumed after complete re‐epithelialization, usually 5–7 days after laser therapy, to reduce discomfort and post‐inflammatory hyperpigmentation.

TXA intradermal 10 mg/mL with a 30 G needle. Microinjections were administered intradermally at a depth of 1–2 mm, with injection points approximately 1 cm apart. A volume of 0.05–0.1 mL was administered per point, with a maximum total dose of 50 mg per session. Treatment was performed in three sessions at 3‐week intervals. Light topical anesthesia was used when needed. The desired endpoint was modest wheal formation with minimal bleeding.

### 2.8 Safety Assessment

Safety was assessed throughout the study. Before receiving oral TXA, patients were screened for thromboembolic risk. At each visit, participants were asked about headache, gastrointestinal discomfort, procedure‐site pain or burning, erythema, edema, post‐inflammatory hyperpigmentation, and any other adverse events. Adverse events were recorded as counts and percentages in each treatment group and are summarized in Table 1.

**TABLE 1:** Safety outcomes during the study.

Adverse event	Oral TXA (*n* = 15)	Er:YAG laser (*n* = 15)	ID‐TXA (*n* = 15)
Mild headache	2 (13.3%)	0 (0%)	0 (0%)
Mild gastrointestinal discomfort	3 (20.0%)	0 (0%)	0 (0%)
Transient erythema	0 (0%)	5 (33.3%)	8 (53.3%)
Transient edema	0 (0%)	3 (20.0%)	4 (26.7%)
Pain/burning at procedure site	0 (0%)	2 (13.3%)	10 (66.7%)
Post‐inflammatory hyperpigmentation	0 (0%)	0 (0%)	0 (0%)
Thromboembolic event	0 (0%)	0 (0%)	0 (0%)
Treatment discontinuation due to adverse events	0 (0%)	0 (0%)	0 (0%)

*Note:* Values are presented as *n* (%).

Abbreviations: ID, intradermal; TXA, tranexamic acid.

### 2.9 Statistical Analysis

Statistical analysis was performed using SPSS version 24. Quantitative data are presented as mean ± standard deviation, and categorical variables are presented as frequencies and percentages. Normality was assessed using the Shapiro–Wilk test. Between‐group comparisons were performed using one‐way ANOVA or Kruskal‐Wallis tests as appropriate, with Bonferroni adjustment for pairwise comparisons. Within‐group pre‐ and post‐treatment changes were evaluated using paired tests as appropriate. Pearson or Spearman correlation tests were used to analyze associations between variables. Post‐treatment MASI scores were compared using ANOVA, as applicable. Statistical significance was set at *p* < 0.05.

## 3 Results

### 3.1 Baseline Characteristics

Baseline demographic variables were comparable across the three treatment groups. No significant differences were observed in age (*p* = 0.414) or sex distribution (*p* = 0.347), indicating satisfactory baseline homogeneity (Table 2).

**TABLE 2:** Baseline characteristics of the treatment groups.

Characteristic	Oral TXA (*n* = 15)	Er:YAG laser (*n* = 15)	ID‐TXA (*n* = 15)	*p*‐value*
Age, years	46.27 ± 5.1	42.73 ± 6.5	44.27 ± 6.4	0.414
Pairwise age comparisons**	I vs. II = 0.188	II vs. III = 0.565	I vs. III = 0.453	
Female sex, *n* (%)	14 (93.3)	14 (93.3)	15 (100)	0.347
Male sex, *n* (%)	1 (6.7)	1 (6.7)	0 (0)	

Abbreviations: ID, intradermal; TXA, tranexamic acid.

* *p*‐value for between‐group comparison using one‐way ANOVA for continuous variables and chi‐square/Fisher exact test for categorical variables as appropriate.

** Pairwise comparisons after Bonferroni adjustment. Group I = oral TXA; Group II = fractional Er:YAG laser; Group III = intradermal TXA**.

### 3.2 MASI Outcomes

**FIGURE 1:**
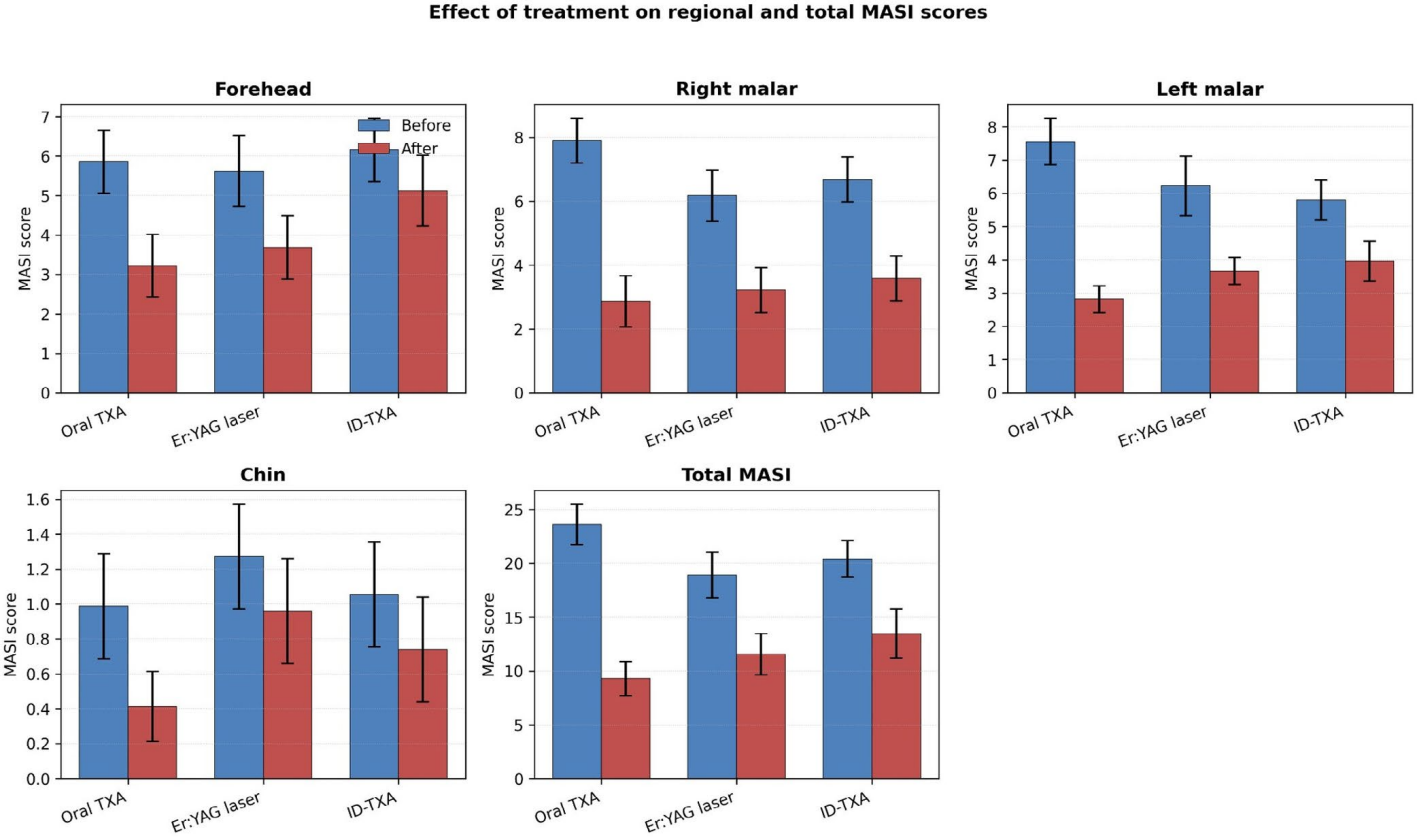
Effect of treatment on regional and total MASI scores. Bars represent mean ± standard deviation before and after treatment for forehead, right malar, left malar, chin, and total MASI scores in the oral TXA, fractional Er:YAG laser, and intradermal TXA groups.

**FIGURE 2:**
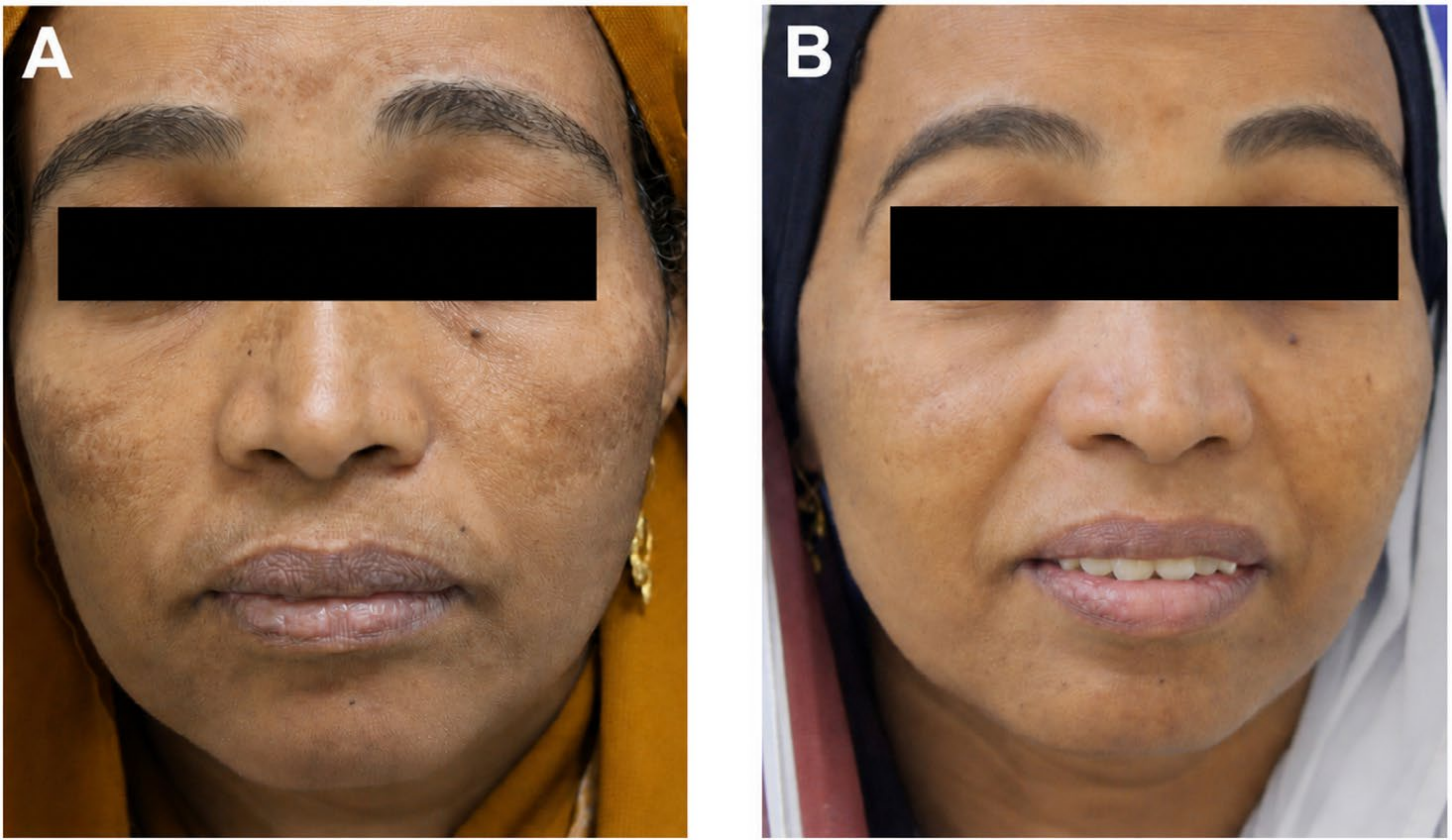
Clinical response to oral tranexamic acid under standardized lighting conditions. Representative standardized clinical photographs showing refractory melasma before treatment with oral TXA combined with topical hydroquinone (A) and clinical improvement after treatment (B).

**FIGURE 3:**
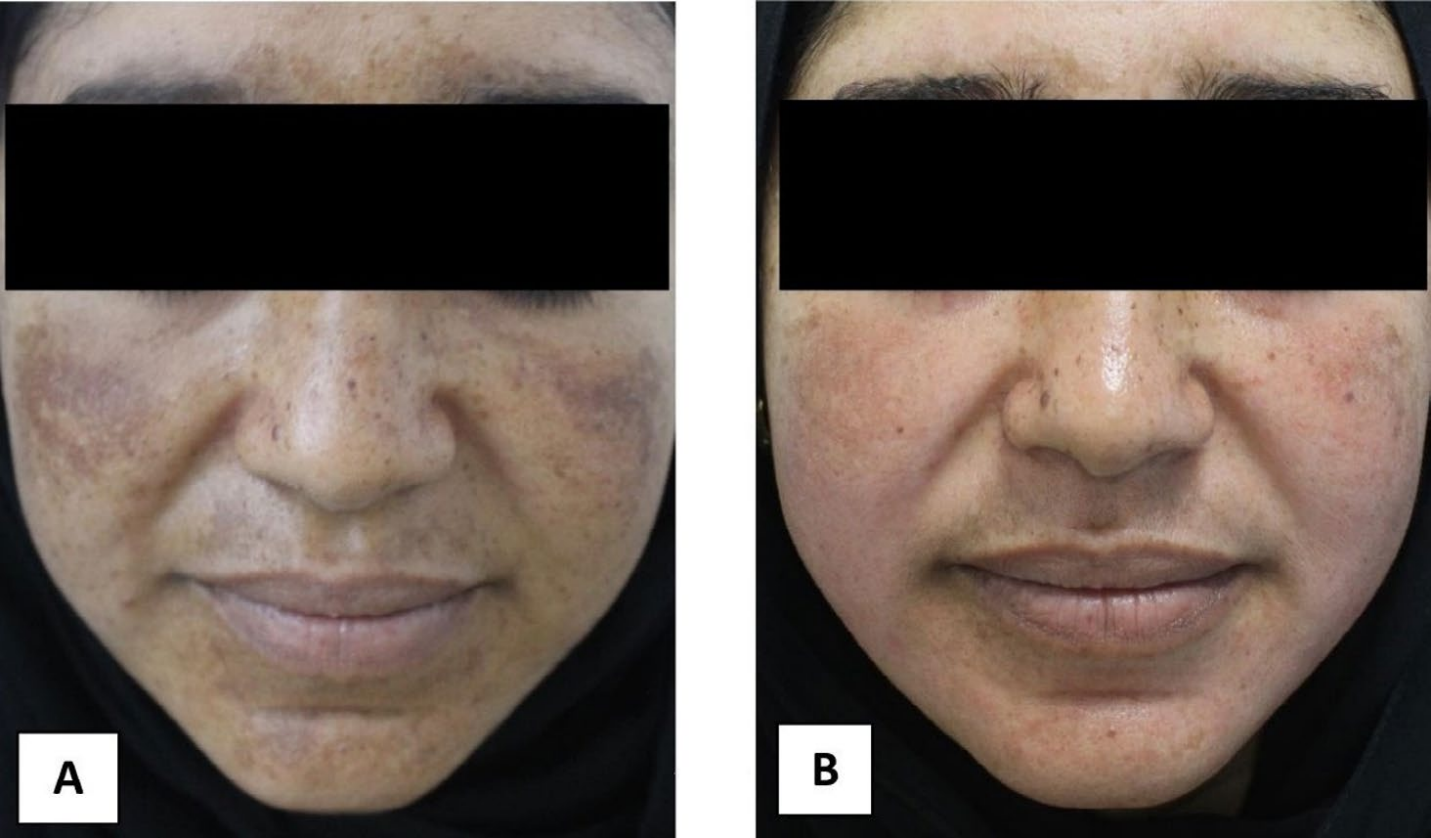
Clinical response to fractional Er:YAG laser under standardized lighting conditions. Representative standardized clinical photographs showing refractory melasma before treatment with fractional Er:YAG laser combined with topical hydroquinone (A) and clinical improvement after treatment (B).

**FIGURE 4:**
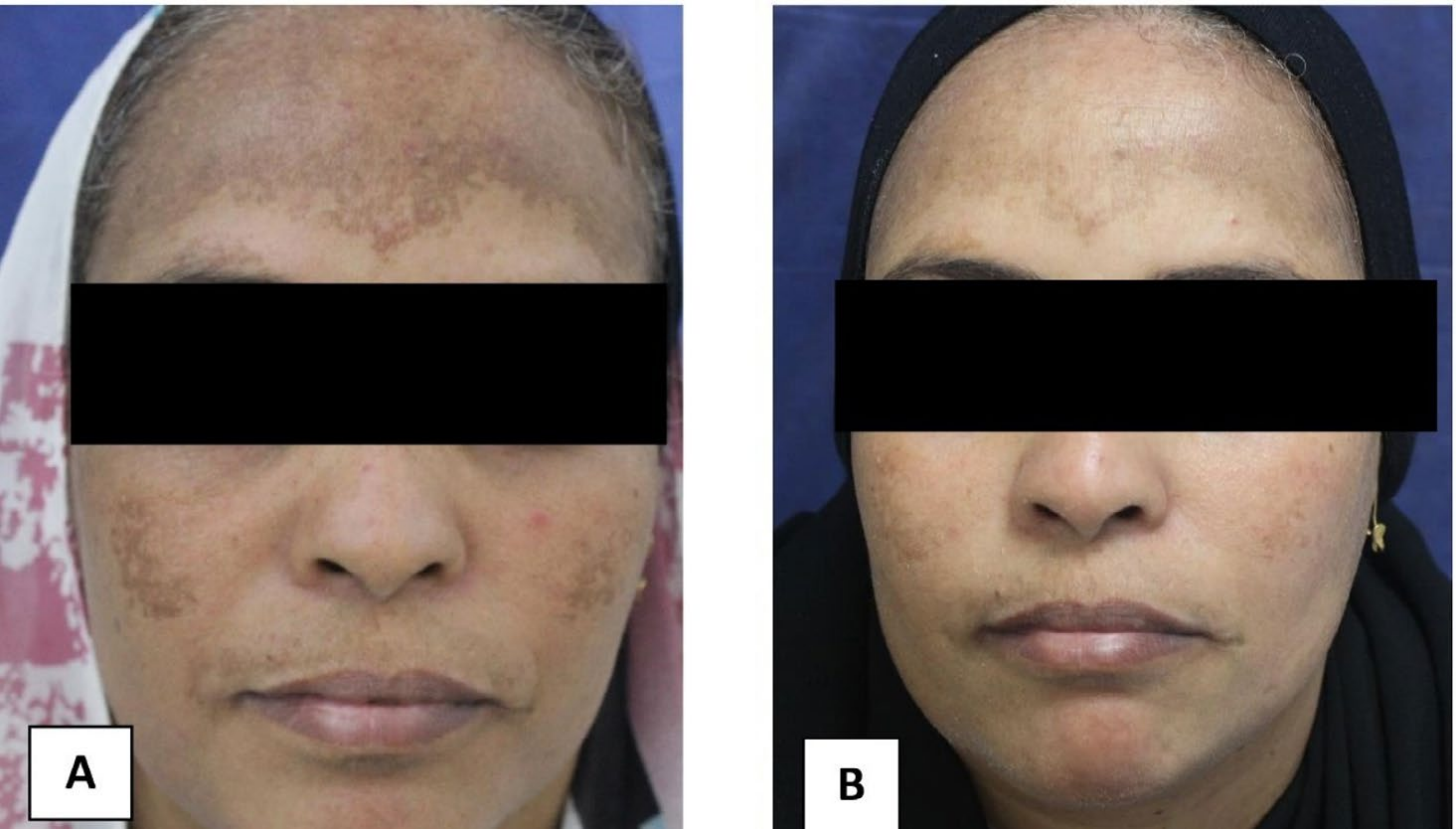
Clinical response to intradermal tranexamic acid under standardized lighting conditions. Representative standardized clinical photographs showing refractory melasma before treatment with intradermal TXA combined with topical hydroquinone (A) and clinical improvement after treatment (B).

All treatment arms showed improvement in regional and total MASI scores after treatment (Table 3; Figure 1). In total MASI, oral TXA achieved the greatest mean reduction (23.61 ± 1.9 to 9.30 ± 1.6), followed by fractional Er:YAG laser (18.91 ± 2.1 to 11.56 ± 1.9) and intradermal TXA (20.41 ± 1.7 to 13.48 ± 2.3). The percentage improvement differed significantly among groups, with the highest response in the oral TXA group, followed by the Er:YAG laser group and the intradermal TXA group. Representative standardized clinical photographs demonstrating treatment response are shown in Figures 2, 3, 4.

**TABLE 3:** Regional and total MASI outcomes before and after treatment.

MASI area	Treatment group	Before, mean ± SD	After, mean ± SD	Within‐group *p* ***	% change, mean ± SD	Between‐group *p* for % change*
Forehead	Oral TXA	5.86 ± 0.8	3.22 ± 0.8	0.003	45.0 ± 5.7	0.037
	Er:YAG laser	5.62 ± 0.9	3.68 ± 0.8	0.001	34.5 ± 4.3	
	ID‐TXA	6.16 ± 0.8	5.12 ± 0.9	0.012	17.0 ± 3.1	
Right malar	Oral TXA	7.9 ± 0.7	2.86 ± 0.8	< 0.001	64.0 ± 9.1	0.042
	Er:YAG laser	6.18 ± 0.8	3.22 ± 0.7	< 0.001	48.0 ± 3.9	
	ID‐TXA	6.68 ± 0.7	3.58 ± 0.7	0.001	46.5 ± 4.1	
Left malar	Oral TXA	7.56 ± 0.7	2.82 ± 0.4	< 0.001	62.5 ± 6.5	0.034
	Er:YAG laser	6.22 ± 0.9	3.66 ± 0.4	< 0.001	41.0 ± 3.8	
	ID‐TXA	5.8 ± 0.6	3.96 ± 0.6	0.040	31.5 ± 2.9	
Chin	Oral TXA	0.987 ± 0.3	0.413 ± 0.2	0.024	58.0 ± 4.7	0.027
	Er:YAG laser	1.27 ± 0.3	0.96 ± 0.3	0.038	24.5 ± 1.6	
	ID‐TXA	1.05 ± 0.3	0.74 ± 0.3	0.061	30.0 ± 2.1	
Total MASI	Oral TXA	23.6 ± 1.9	9.3 ± 1.6	< 0.001	60.5 ± 6.2	0.002
	Er:YAG laser	18.9 ± 2.1	11.6 ± 1.9	< 0.001	38.5 ± 2.9	
	ID‐TXA	20.4 ± 1.7	13.5 ± 2.3	0.001	19.0 ± 1.8	

Abbreviations: ID, intradermal; MASI, Melasma Area and Severity Index; SD, standard deviation; TXA, tranexamic acid.

* Between‐group *p*‐value for percentage change.

** Bonferroni‐adjusted pairwise comparisons for percentage change: Forehead, I vs. II = 0.356, II vs. III = 0.038, I vs. III = 0.033; Right malar, I vs. II = 0.048, II vs. III = 0.412, I vs. III = 0.043; Left malar, I vs. II = 0.039, II vs. III = 0.046, I vs. III = 0.028; Chin, I vs. II = 0.025, II vs. III = 0.464, I vs. III = 0.037; Total MASI, I vs. II = 0.021, II vs. III = 0.031, I vs. III < 0.001.

*** Within‐group pre‐post comparison. Group I = oral TXA; Group II = fractional Er:YAG laser; Group III = intradermal TXA.

### 3.3 Safety Outcomes

No thromboembolic events, serious adverse events, post‐inflammatory hyperpigmentation, or treatment discontinuations occurred during the study. In the oral TXA group, mild headache occurred in 2 patients (13.3%) and mild gastrointestinal discomfort occurred in 3 patients (20.0%). In the Er:YAG laser group, transient erythema occurred in 5 patients (33.3%), transient edema in 3 patients (20.0%), and pain/burning at the procedure site in 2 patients (13.3%). In the ID‐TXA group, transient erythema occurred in 8 patients (53.3%), transient edema in 4 patients (26.7%), and pain/burning at the procedure site in 10 patients (66.7%). All adverse events were mild, transient, and managed conservatively.

## 4 Discussion

Melasma is a chronic and relapsing pigmentary disorder with multifactorial pathogenesis. In this randomized clinical trial, oral TXA, fractional Er:YAG laser, and intradermal TXA, each combined with topical 4% hydroquinone, improved MASI scores in patients with refractory melasma. Oral TXA produced the greatest short‐term improvement, followed by fractional Er:YAG laser, whereas intradermal TXA showed a comparatively lower response.

The favorable response with oral TXA may reflect systemic inhibition of the plasminogen‐plasmin pathway, with subsequent suppression of ultraviolet‐induced melanocyte activation, inflammatory mediator release, angiogenesis, and melanogenesis [9, 10]. This finding is consistent with previous studies reporting meaningful improvements in melasma with oral TXA [12, 13, 14, 15, 16, 17]. In the present trial, oral TXA produced the largest percentage reduction in total MASI and was generally well tolerated. Nevertheless, careful patient selection remains essential because TXA should be avoided in patients with thrombotic risk factors.

The fractional Er:YAG laser ranked second in efficacy. Its effect may be related to controlled epidermal ablation, removal of melanin‐containing keratinocytes, epidermal renewal, and dermal remodeling [11]. These results are compatible with previous reports supporting the Er:YAG laser for melasma, particularly when combined with topical depigmenting treatment [18, 19]. However, laser therapy should be used cautiously in darker skin types because irritation and post‐inflammatory hyperpigmentation may occur.

Intradermal TXA produced significant but comparatively lower improvement. Although localized TXA delivery may reduce systemic exposure, its effect may be limited by uneven drug distribution, insufficient control of systemic triggers, and injection‐related inflammation. Previous studies have reported variable outcomes with intradermal TXA, indicating that concentration, injection technique, treatment interval, and number of sessions may influence efficacy [10, 20, 21, 22].

Safety findings were consistent with the expected tolerability profile of the three interventions. Oral TXA was associated only with mild headache and gastrointestinal discomfort, while procedure‐related erythema, edema, and pain/burning were more frequent after laser and intradermal TXA interventions. Importantly, no thromboembolic events, post‐inflammatory hyperpigmentation, serious adverse events, or treatment discontinuations were observed; however, the sample size and short follow‐up limit conclusions regarding rare or delayed adverse events.

The absence of a hydroquinone‐only control arm should be considered when interpreting the results. Because all groups received hydroquinone and photoprotection, this study cannot determine the independent contribution of hydroquinone or fully exclude differential response to hydroquinone among participants. The findings should therefore be interpreted as a comparison of three adjunctive strategies added to a shared topical background regimen. Future trials should consider a hydroquinone‐only or vehicle‐controlled background arm when clinically and ethically feasible.

The 3‐month follow‐up period is another important limitation. This interval is sufficient for short‐term MASI assessment after completion of treatment but is inadequate to determine durability, relapse, rebound pigmentation, delayed adverse events, or long‐term safety. Future randomized trials should include extended follow‐up, preferably 6–12 months or longer, to assess recurrence rates and sustained safety.

To reduce redundancy, the revised manuscript consolidates regional and total MASI outcomes into one summary table and one quantitative figure with error bars, while retaining only three representative standardized clinical photographs to illustrate the response of each treatment arm.

In conclusion, all three adjunctive treatments improved refractory melasma when combined with topical hydroquinone and photoprotection. Oral TXA produced the greatest short‐term improvement, whereas fractional Er:YAG laser and intradermal TXA may be useful alternatives in selected patients. Larger multicenter trials with longer follow‐up and objective colorimetric or quality‐of‐life outcomes are needed to confirm durability, recurrence rates, and long‐term safety.

## 5 Limitations

This study has several limitations. First, the sample size was relatively small, with 15 patients in each group, which may limit generalizability. Second, follow‐up was limited to 3 months; therefore, long‐term efficacy, recurrence, rebound pigmentation, delayed adverse events, and sustained safety could not be fully evaluated. Third, the absence of a hydroquinone‐only control group prevents isolation of the independent clinical effect of hydroquinone and means the trial compares adjunctive treatments added to a shared hydroquinone background. Fourth, objective tools such as colorimetry, dermoscopy, and validated quality‐of‐life scores were not used. Finally, clinical photographs were discarded as outcome figures because photographic lighting and acquisition conditions were not sufficiently standardized for formal evaluation. Larger multicenter studies with longer follow‐up and objective assessment tools are recommended.

## Author Contributions

Conceptualization: Eisa Mohamed Hegazy, Mahmoud Ahmed Ali, Moustafa Adam El Taieb, Ebtehal Alaa Kotop. Methodology: Eisa Mohamed Hegazy, Mahmoud Ahmed Ali, Mohamed Amer Ahmed Abdellatif. Investigation: Heba Allah Mohamed Mustafa Mohamed, Mohamed Amer Ahmed Abdellatif, Ebtehal Alaa Kotop. Data curation: Heba Allah Mohamed Mustafa Mohamed, Mohamed Amer Ahmed Abdellatif, Eisa Mohamed Hegazy. Formal analysis: Eisa Mohamed Hegazy, Mahmoud Ahmed Ali, Moustafa Adam El Taieb. Resources: Eisa Mohamed Hegazy, Mahmoud Ahmed Ali, Mohamed Amer Ahmed Abdellatif, and the participating departments. Visualization: Eisa Mohamed Hegazy, Mahmoud Ahmed Ali, Ebtehal Alaa Kotop. Writing – original draft: Eisa Mohamed Hegazy, Mahmoud Ahmed Ali. Writing – review and editing: Eisa Mohamed Hegazy, Moustafa Adam El Taieb, Heba Allah Mohamed Mustafa Mohamed, Mohamed Amer Ahmed Abdellatif, Ebtehal Alaa Kotop, Mahmoud Ahmed Ali. Project administration: Eisa Mohamed Hegazy, Mahmoud Ahmed Ali. Supervision: Moustafa Adam El Taieb, Mahmoud Ahmed Ali. Funding acquisition: Not applicable; no external funding.

## Funding

No external funding was received for this study.

## Disclosure

Guarantor: Eisa Mohamed Hegazy, MD.

## Ethics Statement

Approved by the Institutional Review Board of the Faculty of Medicine, Aswan University, Aswan, Egypt (IRB code: 932/6/24).

## Consent

Written informed consent for participation and publication of de‐identified clinical data and images, where applicable, was obtained from all participants.

## Conflicts of Interest

The authors declare no conflicts of interest.

## Data Availability

The data that support the findings of this study are available on request from the corresponding author. The data are not publicly available due to privacy or ethical restrictions.
